# Masked emotions: does children’s affective state influence emotion recognition?

**DOI:** 10.3389/fpsyg.2024.1329070

**Published:** 2024-06-19

**Authors:** Maria Eirini Mastorogianni, Styliani Konstanti, Ioanna Dratsiou, Panagiotis D. Bamidis

**Affiliations:** ^1^MSc in Learning Technologies-Education Sciences, School of Early Childhood Education, School of Electrical and Computer Engineering, School of Medicine, Aristotle University of Thessaloniki (AUTH), Thessaloniki, Greece; ^2^Medical Physics and Digital Innovation Laboratory, School of Medicine, Faculty of Health Sciences, Aristotle University of Thessaloniki (AUTH), Thessaloniki, Greece

**Keywords:** emotion recognition, face masks, COVID-19, affective state, elementary children, affective priming

## Abstract

**Introduction:**

Facial emotion recognition abilities of children have been the focus of attention across various fields, with implications for communication, social interaction, and human behavior. In response to the COVID-19 pandemic, wearing a face mask in public became mandatory in many countries, hindering social information perception and emotion recognition. Given the importance of visual communication for children’s social-emotional development, concerns have been raised on whether face masks could impair their ability to recognize emotions and thereby possibly impact their social-emotional development.

**Methods:**

To this extent, a quasiexperimental study was designed with a two-fold objective: firstly, to identify children’s accuracy in recognizing basic emotions (anger, happiness, fear, disgust, sadness) and emotional neutrality when presented with faces under two conditions: one with no-masks and another with faces partially covered by various types of masks (medical, nonmedical, surgical, or cloth); secondly, to explore any correlation between children’s emotion recognition accuracy and their affective state. Sixty-nine (69) elementary school students aged 6-7 years old from Greece were recruited for this purpose. Following specific requirements of the second phase of the experiment students were assigned to one of three (3) distinct affective condition groups: Group A-Happiness, Group B-Sadness, and Group C-Emotional Neutrality. Image stimuli were drawn from the FACES Dataset, and students’ affective state was registered using the self-reporting emotions-registration tool, AffectLecture app.

**Results:**

The study’s findings indicate that children can accurately recognize emotions even with masks, although recognizing disgust is more challenging. Additionally, following both positive and negative affective state priming promoted systematic inaccuracies in emotion recognition. Most significantly, results showed a negative bias for children in negative affective state and a positive bias for those in positive affective state.

**Discussion:**

Children’s affective state significantly influenced their emotion recognition abilities; sad affective states led to lower recognition overall and a bias toward recognizing sad expressions, while happy affective states resulted in a positive bias, improving recognition of happiness, and affecting how emotional neutrality and sadness were actually perceived. In conclusion, this study sheds light on the intriguing dynamics of how face masks affect children’s emotion recognition, but also underlines the profound influence of their affective state.

## 1 Introduction

There’s minimal consensus on a response, leading to uncertainty regarding the boundaries of emotion and its origins and effects (James, 1884). Russell (2003) prompts a series of thought-provoking inquiries regarding the nature of emotions; What is an emotion? Are emotions to be conceptualized as brain modes, actions or action tendencies, reflexes, instincts, attitudes, cognitive structures, motives, sensations, or feelings? Are they biologically fixed modules (and hence reducible to biology) or socially constructed roles (and reducible to sociology)? Discrete categories or bipolar dimensions? Cognitive, precognitive, or postcognitive? Exploring the nature of emotions extends beyond theoretical definitions to practical applications in social interactions. The importance of non-verbal communication and the capacity to comprehend the emotions of others are significant aspects of social interaction. It facilitates coordination with others, enhances communication in general, and serves as a crucial element of the “affective glue” that binds individuals in dyadic interactions (Niedenthal and Brauer, 2012). However, individuals often make inaccurate judgments regarding others’ emotional expressions, leading to misinterpretations of their emotional states, which can negatively impact social interactions (Elfenbein et al., 2007). In this context, it is crucial to reflect on the significance of accuracy and inaccuracy in perceiving facial expressions of emotion. Recognizing basic emotions through facial expressions serves as a foundational aspect of emotion recognition, and accuracy in emotion decoding (EDA) plays a vital role in regulating personal and social relationships (Manstead et al., 1999). However, despite over 40 years of consecutive research in facial expression, scientists are still in disagreement regarding whether emotions are accurately conveyed through facial expressions and the exact information conveyed by these expressions remains a subject of debate (Gaspar et al., 2014).

As discussions continue about the accuracy of emotions conveyed through facial expressions, understanding how these debates have evolved historically provides valuable insights. Darwin’s (1872/1965) seminal work “The expression of Emotions in Man and Animals” introduced the concept that expressive movements have evolved over time and proposed the existence of universal patterns of expression that transcend species boundaries. Wundt (1896) highlighted historical debates over the term “basic emotions,” but various definitions in literature generally agree that facial expressions are composed of micro-motor movements, aiding in deducing an individual’s emotional state. In the 1960s and 1970s, there was a revival of interest in the evolution of facial expressions, particularly focusing on the evolutionary patterns observed in primate facial expressions. This renewed interest was evident in the works of researchers such as Andrew (1963), Chevalier-Skolnikoff (1982), and Van Hooff (2017). At the same time, American psychologists revisited the evolution of human expression and generated vast amounts of data supporting the existence of a core set of universal facial expressions corresponding to fundamental emotions. Central to this line of thinking was the identification of a few facial expressions which were proposed to be universal signals for certain basic emotions, more specifically the methods that lead to that identification (Gaspar et al., 2014). Ekman’s significant research on facial expressions has revealed that emotions like anger, disgust, fear, happiness, sadness, and surprise are universally recognized by both literate and preliterate cultures worldwide (Ekman, 1971; Ekman, 1992). The facial affect program, known as the *Facial affects program* (Ekman et al., 1969; Ekman, 1971, 1973; Izard, 1971), proposed that upon the elicitation of particular emotional states, there is a universally automatic activation of specific facial muscles, integral to the emotional experience itself.

Hess and Hareli (2015) work on facial emotion recognition provides useful insights on the two main approaches to the EDA: *pattern matching* and *perspective taking*. Pattern matching involves linking specific features of facial expressions to particular emotions (Buck, 1984). For instance, an observer may associate upturned corners of the mouth with smiles and lowered eyebrows with frowns, thereby inferring happiness or anger, respectively. Consequently, participants are often presented with contextless faces, sometimes with hairlines removed, to highlight these informative features. In this task, observers are tasked with labeling perceived facial configurations without considering contextual or expresser-related factors. This cognitive process resembles the approach employed by facial expression recognition software and does not necessarily rely on the broader social knowledge of the observer. Another process involved in emotion perception is perspective taking, which relies on the observer’s social knowledge. Perspective taking can help justify observed expressions retroactively, such as when we try to understand why a friend became angry over a seemingly harmless comment. It can also aid in predicting the likely expression of someone experiencing an event. For instance, upon learning that someone received good news, one might predict that the person is now happy rather than disappointed. Additionally, the social group membership of the expresser provides another source of information. People often hold stereotypes about members of different groups, which can influence emotional perception (Philippot et al., 1999). We suggest that in most situations, observers utilize perspective taking and their accumulated knowledge of emotions to actively interpret expressions within their context. This process entails engagement with social knowledge, where participants employ social cognition and theory of mind to use pattern matching for deducing emotional states based on facial expressions in context.

According to Hess and Hareli (2015) the degree to which context influences emotion perception varies. Contextual effects on emotion perception are contingent upon the clarity of the cues used to identify the emotion. Strong and prototypical expressions of emotion, such as those commonly utilized in classic emotion recognition studies, are generally less susceptible to contextual influences. Conversely, weaker, and more ambiguous expressions, frequently encountered in everyday life, often necessitate additional contextual knowledge for accurate interpretation. Similarly, when multiple emotion cues are present, each suggesting different emotions, interpretation may require additional context. In cases where cues provide no specific emotional information, such as in neutral facial expressions, context becomes the primary determinant of identification. On the other hand, certain emotions may serve as potent signals that can be interpreted relatively independently of context.

Recognition of emotions appears to be influenced by various factors and the role of one’s own affective state in emotion recognition remains a pertinent question. Researchers have explored the relationship between one’s affective state and their capacity to recognize emotions in others, although they have not always arrived at a consensus. Mood-congruity theories, as posited by Bower (1981) and Schwarz (1990), propose that an individual’s affective state influences cognitive processes such as memory encoding and social judgments. Specifically, these theories suggest that individuals experiencing negative moods or depressive states exhibit enhanced recall of negative stimuli and tend to make more negative social judgments, indicative of a negative bias. Conversely, positive moods are associated with heightened recall of positive stimuli and more positive social judgments, reflecting a positive bias. When applied to emotion recognition, these theories imply that individuals in sad or depressive states may demonstrate enhanced recognition of sad facial expressions compared to happy ones, while those in happy moods may exhibit superior recognition of happy facial expressions relative to sad ones. Thus, mood-congruity theories offer a framework for understanding how emotional states influence the perception and interpretation of facial expressions, contributing to our understanding of the interplay between affective experiences and social cognition (Drace et al., 2010). When considering the role of one’s own internal states, we must account for the effect of the mirror neuron system (MNS) in understanding others (Juckel et al., 2018). Iacoboni (2008) describes MNS as a pre-reflective, automatic mechanism of mirroring which consists of the activation of the same neurons when doing an action and when observing it. For instance, a depressive person needs happier facial expressions to label them correctly (Mergl et al., 2005). Moreover, evaluations are easier/faster, when one’s own mood and contextual information, as well as parts of the emotional pattern in the face, are congruent (Lindquist et al., 2012). In conclusion, experiencing a negative mood or experiencing depressive symptoms has been found to enhance the retrieval of negative information and increase the likelihood of forming negative judgments towards others, commonly referred to as a negative bias (Mast, 2010).

For instance, support for the congruity hypothesis has predominantly been demonstrated in studies with adults. Mast (2010) demonstrated a negative bias among adults in sad moods, with these participants showing a heightened ability to recognize sad facial expressions compared to happy ones. Conversely, adults in happy moods did not consistently exhibit a superior recognition of happy facial expressions, though the trends were in that direction (Mast, 2010). More recently, Trilla et al. (2021) investigated how adults project their own affective states onto the interpretation of others’ emotional expressions. They found that participants were more likely to classify facial expressions as happy when they reported feeling happy themselves, as opposed to when they felt sad. In contrast, Chepenik et al. (2007) revealed a general decline in emotion recognition performance among healthy individuals in sad moods compared to a control group in a neutral mood.

Although mood congruity effects on emotion recognition have been reported extensively in adults, studies investigating these effects in children younger than 10 years are limited and primarily consist of older research. Studies investigating the mood congruity effect in children, which examine whether individuals remember material better when it matches their mood, have generally yielded negative results (Potts et al., 1986; Forgas et al., 1988). However, Nasby and Yando (1982) achieved some success. In their initial experiment, they observed a mood effect during the encoding phase for material with a positive emotional tone: a happy mood enhanced recall, while a sad mood hindered recall of positive adjectives. Interestingly, this effect was not observed for negative items. Moreover, during retrieval, the mood congruity effect was asymmetrical: a happy mood facilitated recall of positive material, but no such effect was found for a sad mood when compared to a neutral control group. In the realm of social judgment among children, the findings are similarly varied. Four notable studies with conflicting results should be mentioned. Forgas et al. (1988) conducted a study where children were presented with information about two target children and asked to judge them on positive and negative personal characteristics. Interestingly, happy children tended to provide more extreme judgments, both positive and negative, but no evidence of mood congruity was observed. Conversely, Duncan et al. (1985) found no support for mood congruity when children’s mood was examined in relation to judging facial expressions. However, Terwogt et al. (1991) demonstrated an effect of positive mood on a task involving judgment of ambiguous facial expressions. Similarly, Stegge et al. (1995) found a symmetrical effect of mood induction, where children 6 and 7 years old labeled more drawings as happy, compared to sad children.

There are also notable differences between the findings in adults and children. In adults, there is substantial support for the mood congruity effect, with several studies demonstrating a bias in emotional recognition and memory retrieval based on one’s own mood. Conversely, studies in children younger than 10 years have generally produced mixed or negative results when attempting to demonstrate mood congruity effects. While some studies in children have shown limited evidence of mood congruity effects, overall, the findings are less consistent compared to those in adults. In children, the effects of controlled processing may be less apparent, because effective strategies of mood repair are learned in the course of development. Thus, in young children, one expects more symmetrical effects of positive and negative mood states (Isen, 1984). The inconsistencies among studies in children may stem from methodological differences, such as variations in experimental design and measures used to assess mood congruity effects. Additionally, developmental factors may play a role, as children’s cognitive and emotional processing abilities are still developing and may differ from those of adults.

Children’s ability to comprehend others’ affective state, self-regulation, and the appropriate expression of emotion are closely tied to interpersonal relationships and social behavior (Bonti, 2013). Developmental changes do indeed occur from infancy to adulthood, as an individual’s social surroundings gradually expand in both capacity and complexity (Tonks et al., 2007; Barrett et al., 2019). While the recognition of facial emotions significantly improves between 6 and 15 years of age and adulthood (Montirosso et al., 2015), the early stages of childhood are recognized as a pivotal period for the maturation of emotional comprehension and processing (Denham et al., 2003; Compas et al., 2014). Moreover, it seems that children’s proficiency in recognizing various emotional states does not develop all at once. Data suggests, for instance, that positive emotional states are typically acquired first (Boyatzis et al., 1993); specifically, happiness is the first affective state to be accurately recognized, followed by sadness and, later, by anger and surprise (Markham and Adams, 1992). Evidence supports certain affective states, such as happiness and sadness, tend to be more readily recognized by preschoolers compared to emotions like fear and disgust (Gagnon et al., 2014). Their findings further illustrate that emotions typically identified by the upper face include happiness, happiness, surprise, shock, guilt, and sadness, whereas disgust is usually recognized through the middle part of the face. Moreover, emotions primarily recognized from the lower part of the face encompass anger, fear, and surprise. Nevertheless, it’s generally assumed that the lower part of the face plays a pivotal role in recognizing happiness and disgust, while the upper portion is instrumental in the recognition of anger and fear, and both parts are engaged in processing surprise and sadness (Wegrzyn et al., 2017). A study by Guarnera et al. (2015) demonstrated that fear, happiness, and surprise were better recognized when the whole face was presented compared to the eye or mouth areas. Sadness was best identified when the eye area was presented, followed by presentation of the whole face, while the identification of sadness appeared more difficult when the mouth area was presented. Subsequently, neutral emotion was better identified when the mouth area was presented, compared to the whole face or eye area (Guarnera et al., 2015). Numerous studies have consistently highlighted women’s proficiency in decoding emotions, demonstrating an advantage that extends from infancy (Abbruzzese et al., 2019) through adulthood (Thompson and Voyer, 2014; Sullivan et al., 2017; Olderbak et al., 2018). While according to Brody and Hall (2010) children’s recognition of facial expressions tends to show slightly higher accuracy in females.

In light of the existing literature, the emergence of the COVID-19 pandemic has created new incentives for understanding emotion recognition, particularly in children. The outbreak of COVID-19 led to profound changes in various facets of daily life, notably the mandatory use of face masks, as seen in numerous countries during the initial wave of the virus in May 2020 (Brooks et al., 2020). Part of this new reality included the temporary suspension of school and educational institution operations in Greece. Consequently, endeavors were initiated to sustain educational instruction through digital devices and interactive platforms, thereby facilitating remote teaching and learning processes (Sakka et al., 2020). World Health Organization (2020) has issued guidance recommending mask-wearing for all students, staff, and teachers upon the reopening of classrooms. In Greece, as of October 23, 2020, and during the pandemic, universal mask usage was mandated for pre-school and school-age pupils in school halls and within school courtyards (Greece, 2020).

The widespread use of masks in schools has raised concerns about its potential influence on the individual and social lives of children, along with its potential repercussions on their social interactions (Esposito and Principi, 2020) providing an interesting setting for testing whether the ability to read faces can adapt to such an environmental change (Spitzer, 2020). Levitan et al. (2022) explored the extent of the impact of masks on emotion recognition and the perceived intensity of facial expressions. Their study revealed that masks not only impaired the recognition of emotions but also dampened the perceived intensity of positive emotions. These findings align with the broader body of research conducted during the COVID-19 pandemic, which consistently demonstrates the challenges posed by masks in understanding emotions in both adults (Ruba and Pollak, 2020; Ziccardi et al., 2021) and young children (Carbon and Serrano, 2021). Studies spanning 2020 (Gori et al., 2021; Grundmann et al., 2021) and 2021 (Ramachandra and Longacre, 2022) have repeatedly reaffirmed these overarching observations. The difficulty in identifying specific emotions arises from the unique reliance of various emotions on distinct areas of the face. For instance, the mouth and lips assume a critical role in conveying happiness, while the eye region exhibits significant changes when expressing fear or anger, marked by eyelid movements that signify fear or anger, respectively (Gagnon et al., 2014; Wegrzyn et al., 2017). Emotions that predominantly involve the lower part of the face, such as happiness, disgust, and sadness, prove more challenging to discern when individuals wear masks, compared to emotions that rely more on the upper part of the face, like anger and surprise (Langbehn et al., 2020; Bani et al., 2021; Noyes et al., 2021; Tsantani et al., 2022). Notably, neutral expressions tend to be accurately recognized in both masked and unmasked conditions (Noyes et al., 2021).

### 1.1 Rationale of the study

Although debate has arisen over the specific emotions universally recognized, a consensus among diverse studies indicates the existence of basic emotions across cultures. This agreement stems from individuals’ capacity to collectively reach a consensus on affective state when clear facial expressions are presented (Hess, 2014). To this day, and despite conceptual and methodological criticism, basic emotion program continues to develop conceptually and methodologically and is the basis for much “emotion recognition” software (Fernández-Dols and Russell, 2017). Our study aligns with the widespread acceptance and practical implications of the basic emotion approach, as elucidated by Ekman and colleagues (Ekman, 1971; Ekman, 1992). By adhering to this framework, we capitalize on the abundance of empirical evidence supporting the universality of certain emotional expressions, ensuring robustness and generalizability in our findings. Additionally, Ekman’s emphasis on pattern matching in emotion perception resonates with our study objectives, allowing us to elucidate the underlying mechanisms of emotion recognition and contribute to the refinement of existing models. Through our research, we aim to deepen our understanding of facial emotion recognition, advancing this critical area of research and contributing to the cumulative knowledge in the field.

In addition, the relationship between affective state and emotion recognition has been the focus of extensive research. Initially, attention was primarily directed towards investigating this relationship within adult populations, with little consideration given to children. While there is extensive literature on mood congruity in adults, studies focusing on children under 10 years old are limited and primarily based on older research. Given the essential role of emotions in social interactions and the profound impact of the COVID-19 pandemic on children’s daily lives, understanding how their affective state influences their perception of others’ emotions is crucial.

By focusing on these two essential aspects—children’s emotion recognition abilities in the context of face masks and the potential correlation between their affective state and emotion recognition—this study aims to shed light on the role of children’s current affective state in their recognition of emotion in others. Firstly, it endeavors to identify children’s accuracy in recognizing basic emotions (such as anger, happiness, fear, disgust, sadness) and emotional neutrality when presented with faces under two conditions: one with non-covered faces and another with faces partially covered by various types of masks (medical, non-medical, surgical, or cloth). Furthermore, it aims to investigate the relationship between children’s affective state and their proficiency in recognizing emotions accurately. Specifically, it aims to discern how variations in mood-whether happy, sad, or neutral—affect children’s ability to identify happy, sad and neutral facial expressions. The presence of face masks is expected to hinder children’s ability to accurately discern emotions compared to when faces are not covered. Additionally, participants experiencing positive affective states are anticipated to exhibit a positive bias, demonstrating heightened accuracy in recognizing happy faces compared to sad ones. Conversely, those in a negative affective state are expected to display a negative bias, demonstrating superior recognition of sad faces relative to happy ones.

## 2 Materials and methods

### 2.1 Participants and study design

The study was conducted in the first semester of the school year 2020–2021 during the COVID-19 pandemic when general legal obligations to wear masks in Greece were already in action. Participants included sixty-nine (69) children (*n* = 29 boys, *n* = 40 girls) all within the age range of 6 to 7 years old attending the same grade level and recruited from a primary school located in Greece. Children were in their typical academic year, exhibiting no signs of pervasive developmental disorders or any other indications of developmental disabilities. Allocation into groups was necessitated to fulfill the specific requirements of the second phase of the experiment, where students were assigned to one of three distinct affective condition groups: Group A (MoodPriming—Happiness, *n* = 21), Group B (MoodPriming—Sadness, *n* = 26), and Group C (MoodPriming—Emotional Neutrality, *n* = 22). The assignment into groups was based on the pre-existing division of students into three different classes.

Ethical considerations were meticulously addressed throughout the research process. Permission was obtained from the school administration prior to the initiation of the study. Additionally, extensive communication was conducted with the school administration to provide a clear overview of the research objectives and methodology. Furthermore, detailed information was disseminated to the parents of the participating children, outlining the nature of the study and the potential implications. Informed consent forms were distributed to parents, ensuring they were fully aware of their child’s involvement in the research and granting permission for their participation.

Moreover, special emphasis was placed on ensuring the children themselves were fully informed about the experimental procedures. In a child-friendly manner, the research protocol was thoroughly explained to the participating children, empowering them to make autonomous decisions regarding their involvement. It was emphasized that participation was entirely voluntary, and children were assured that they could withdraw from the research at any point without facing any consequences or pressure. Additionally, all ethical guidelines and regulations pertaining to research involving human subjects, especially children, were strictly adhered to. Measures were implemented to guarantee the confidentiality and privacy of the participants’ data and handle any potential issues or concerns raised by parents or the school administration.

### 2.2 Experimental task—stimuli and materials

We consider the design and development of two digital quizzes, referred to as Quiz 1 and Quiz 2. Quiz 1 was developed to evaluate children’s ability to recognize basic emotions in individuals of various age groups. Two conditions were established for the Quiz 1: one with *no masks* and another *with masks*. Quiz 2 was developed to explore the potential correlation between emotion recognition accuracy and children’s current affective state.

#### 2.2.1 Facial emotion recognition in unmasked and masked faces

**Figure 1:**
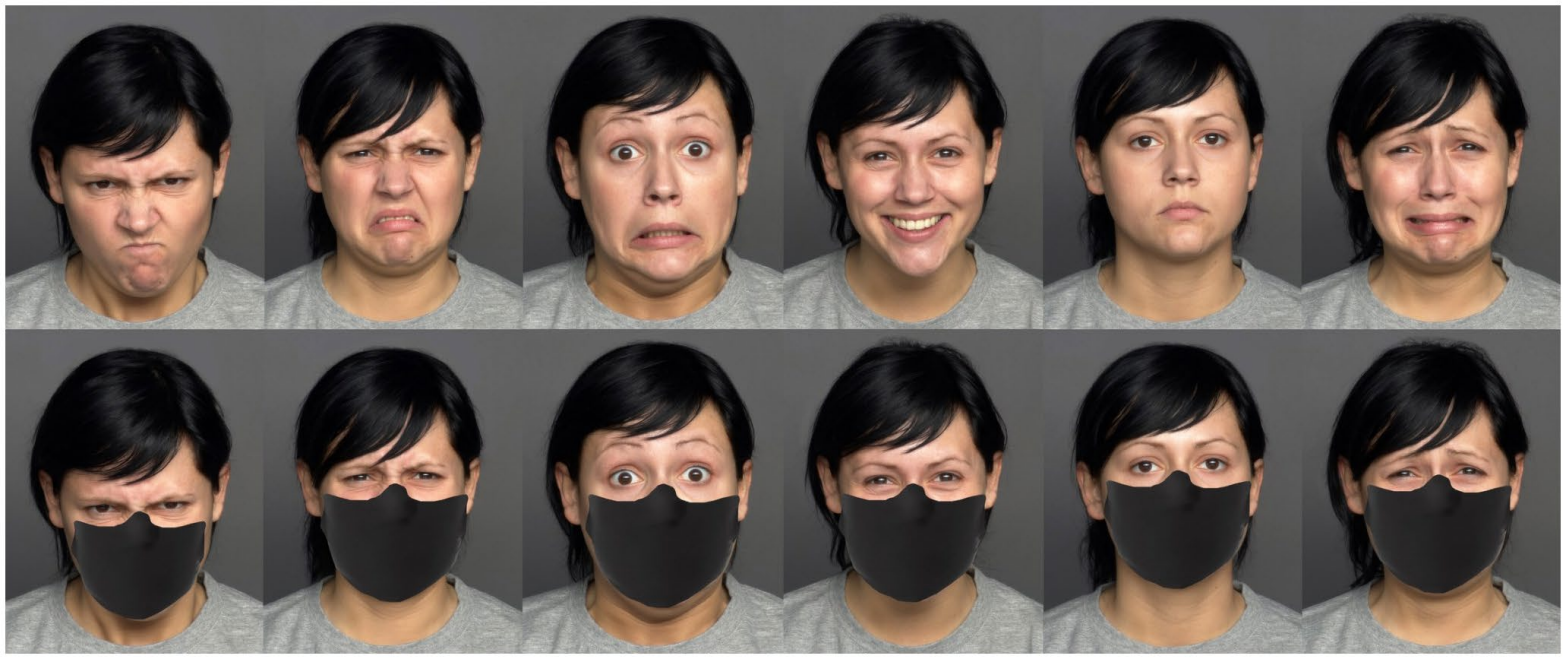
The diagram depicts six different emotional expressions (anger, disgust, fear, happiness, neutrality, and sadness) of an individual, both with and without a face mask. This particular individual was not included in our experimental materials; rather, their image is used here solely for illustrative purposes. The authors extend their gratitude to the Max Planck Institute for generously providing the baseline stimuli (unmasked), sourced from the MPI FACES database (Ebner et al., 2010).

In designing our study, we carefully selected specific emotion expressions to be recognized based on several considerations. For Quiz 1 a selection of images featuring facial expressions conveying fundamental emotions, including happiness, sadness, anger, disgust, or fear, as well as neutral expressions, were curated following Ekman’s facial affect program (Ekman et al., 1969; Ekman, 1971, 1973). The image stimuli were sourced from the FACES Dataset (Ebner et al., 2010) (Figure 1). It is important to mention that we intentionally excluded the emotion of surprise. This decision was made due to limitations within the FACES Dataset, which does not include representations of surprise.

Participants were tasked with identifying and describing facial expressions from the provided images, with six response choices available: anger, disgust, fear, happiness, neutrality, and sadness. Two conditions were established for the task: one with no masks and another with masks. In the mask condition, digital modifications using Adobe Photoshop were made to incorporate face masks covering the mouth and nose regions of the depicted individuals. The stimuli for the task consisted of 13 validated images (*n* = 13) from the FACES platform, specifically featuring frontal images of Caucasians belonging to two distinct facial age groups—a young man, a young woman, and an older woman (comprising 6 men and 7 women).

#### 2.2.2 Affective priming and emotion recognition in masked faces

Conversely, Quiz 2 was structured differently as the aim was to assess participants’ ability to identify the affective state of happiness, sadness, and emotional neutrality in a group of 12 people whose faces were covered with various types of masks, including medical, non-medical, surgical, or cloth masks. This second task comprised 12 questions, each associated with a corresponding image and within these questions, there were four (4) images for each emotion (happiness, sadness, and emotional neutrality). Emotion recognition accuracy was calculated using a traditional accuracy scoring paradigm: responses that matched the target emotion received a score of 1 and responses that did not match received a score of 0 for each of 12 faces.

To ensure the tasks’ appropriateness, we included stimuli with varying emotional intensities. The images chosen for both quizzes depicted varying degrees of emotions rather than solely extreme or prototypical expressions. This approach aimed to mirror real-world scenarios where emotional expressions exhibit nuance and intensity. In addition, to prevent potential memory effects and ensure the validity of our results, we utilized a diverse set of individuals for each task. Quiz 1 featured 13 different images, showcasing individuals across different age groups and genders. Similarly, Quiz 2 comprised 12 images, each portraying individuals with diverse facial characteristics. This diversity aimed to prevent participants from relying on memory cues and avoid biases in emotion recognition.

#### 2.2.3 AffectLecture app

**Figure 2:**
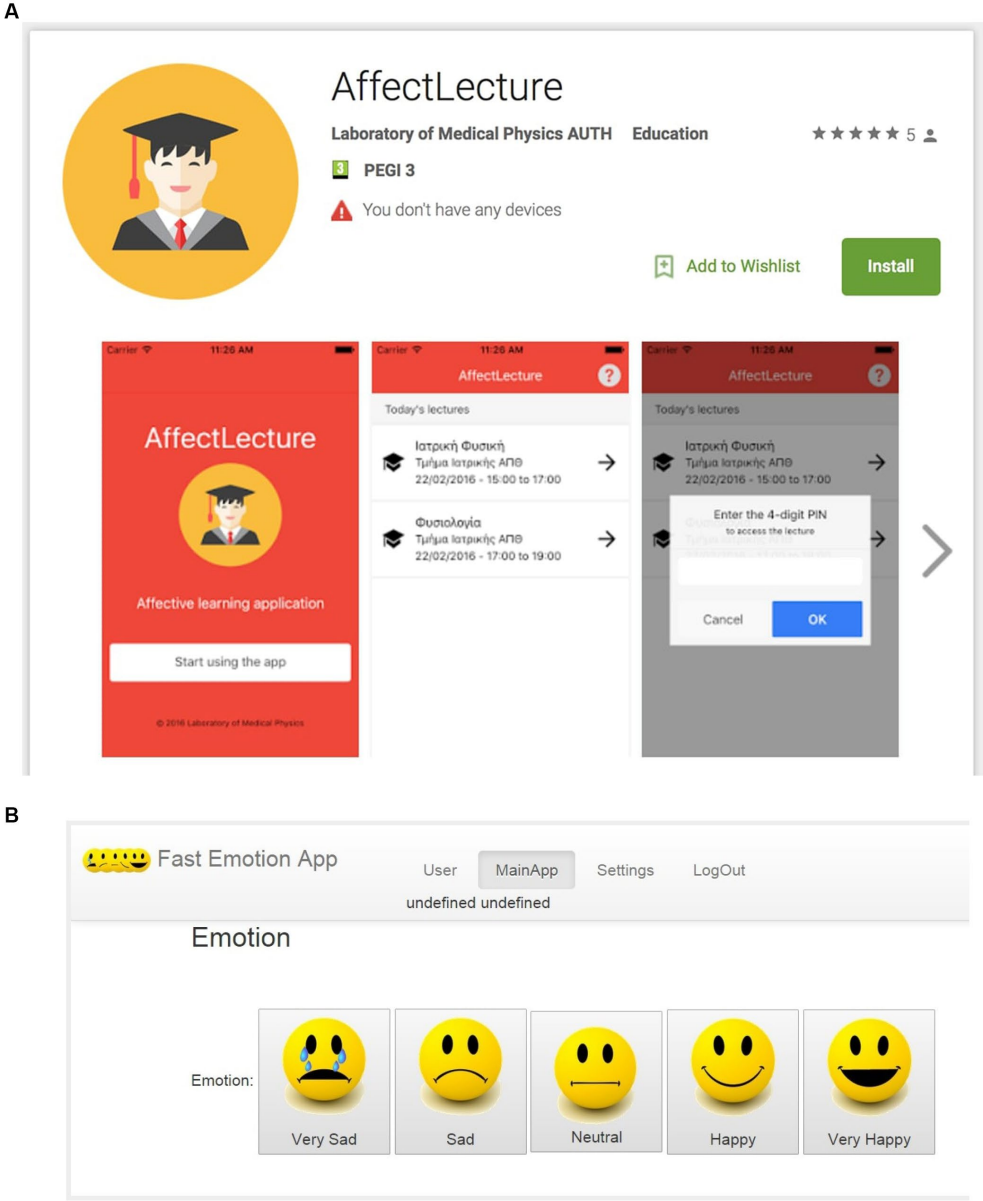
**(A)** AffectLecture App: setting up the content and procedure. **(B)** AffectLecture App: actual face/emotion choices.

The self-reporting, emotions-registering tool AffectLecture app was used before and after the mood priming to explore participants’ affective state and ensure that it was affected by the corresponding movie scene. AffectLecture is a hybrid mobile application designed for self-reporting and tracking emotional states. It utilizes a five-level Likert scale, with “1” representing “very sad” and “5” representing “very happy,” using emoticons for visual representation (Antoniou et al., 2017; Siouli et al., 2020). Originally developed to monitor students’ moods before and after university or classroom lectures, AffectLecture has also been applied to monitor emotional changes in smokers undergoing biofeedback and neurofeedback training, among other developmental/educational protocols (Pandria and Bamidis, 2023). Access to specific sessions is restricted to users attending the session, requiring a 4-digit PIN provided by the instructor who registers the session on the app. Upon entering the correct PIN, users can select the emoticon that best reflects their current mood from a toolbar (Figures 2A,B).

### 2.3 Procedure

Participants engaged in two emotion recognition quizzes, as previously described, denoted as Quiz 1 and Quiz 2. These assessments were meticulously devised and constructed by the research team using Microsoft Forms. The experiment initiated with Quiz 1, where all children individually assessed their emotional recognition abilities by identifying facial expressions in a series of images featuring faces depicting fundamental emotions, namely happiness, sadness, anger, disgust, or fear, alongside neutral expressions under two conditions: one with non-covered faces and another with faces partially covered by various types of masks (medical, non-medical, surgical, or cloth). Afterwards, to explore any correlation between children’s emotion recognition accuracy and their affective state, we primed participants with a movie commonly associated with basic emotions. Specifically, before the Quiz 2 the affective state of the three affective condition groups was elicited through exposure to corresponding scenes from the film “Inside Out,” a 2015 American animated film produced by Pixar Animation Studios.

Group A—Happiness was exposed to notable movie clips that typically elicits joy, and we suggest that it may lead to increased accuracy in identifying happiness from faces partially covered by various types of masks (medical, non-medical, surgical, or cloth). These scenes, spanning from approximately 00:02:33 to 00:07:21 and seamlessly continuing to 00:34:23 to 00:36:00, portray Riley spending quality time with her family, engaging in fun activities, and sharing laughter and smiles. The vibrant, warm colors (reds, oranges, yellows) stimulate happiness, creating an uplifting atmosphere for both Riley and the audience. The first clip closes with Riley enjoying the simple pleasure of sleeping. Following that, the second movie clip opens with Riley waking up. Then, Joy and Sadness meet the Memory Workers, who are in charge of dumping out memory orbs that have faded into the Memory Dump. This clip is also filled with joy and upbeat energy as Riley’s joyful memories associated with the catchy TripleDent Gum jingle are depicted. Joy herself is quite prominent in this scene, reflecting Riley’s happy moments tied to the jingle. It’s a moment that captures the essence of happiness and nostalgia for Riley.

Group B—Sadness, similarly, was exposed to scenes meant to induce feelings of sadness because we suggest that it may lead to increased accuracy in identifying sadness. These scenes span approximately from 01:05:04 to 01:20:00, when Riley experiences moments of disappointment, loss, and sadness. Joy and Bing Bong, Riley’s childhood imaginary friend, have fallen into the Memory Dump and are stuck down there, doomed to be forgotten forever. It’s a poignant moment that explores themes of loss, growing up, and the fading of childhood innocence. The scenes represent sadness, are full of dark, muted and neutral colors, such as gray, brown, and certain shades of blue. The audience is supposed to feel upset.

Group C—Emotional Neutrality watched movie clips meant to maintain emotional neutrality. In the movie clips, both groups get to peer inside the head of Riley, the main character. Five emotions—personified as the characters Anger, Disgust, Fear, Sadness and Joy—struggle for control in her mind. The movie clips span from 00:00:00 to 00:02:34 and 00.18.11 to 00.23.27. In the first scene Riley is presented as a baby. Instrumental piano music and neutral colors (beige, gray, cream) compose a scene that’s neither entirely joyful nor sad. Following that, the second movie clip opens with Riley in a calm state, her emotions are not as active, representing a state of emotional neutrality. There are moments when Riley is at school and at home doing everyday tasks like sleeping and eating dinner with her family. During these scenes, none of her emotions (Joy, Sadness, Anger, Fear, or Disgust) may be prominently featured or driving the narrative. Instead, Riley may be in a state of emotional neutrality, experiencing a sense of calm. This movie clip also highlights the importance of emotional balance and the occasional need for emotional neutrality to navigate complex situations. The audience is supposed to maintain emotional neutrality.

Before and after exposure to these scenes, each individual within every respective group provided self-reported assessments of their affective state via the self-reporting, emotions registering AffectLecture app. Once the desired affective state was effectively induced and ensured within each group, Quiz 2 was initiated. In this quiz, students were tasked with identifying the affective states of happiness, sadness, and emotional neutrality in a series of images depicted individuals with masks. One point was assigned for each correct response, while no points were assigned for incorrect responses. Participants were allowed as much time as needed to make their choice; thus, reaction time was not included as a variable in any analyses.

### 2.4 Statistical analysis

SPSS was used to conduct all the statistical analysis of this study. Upon inspection of the data distributions using histograms and Q-Q plots it was decided that they did not look normally distributed. A Shapiro–Wilk test confirmed this (*p* < 0.001) and thus non-parametric analyses were performed (Morgan et al., 2004) (see Supplementary material, Supplement 1). To assess children’s emotion recognition accuracy under two conditions, we transformed their answers into a binary variable of “success” and “failure” and used not only descriptive statistics but also a binomial test. We also used a binomial test to compare the frequency distribution of participants with that of the population. Pearson Chi-Square statistics were also employed to examine the relationship between children’s emotion recognition and gender. Furthermore, to deeper explore the relationship between children’s facial emotion recognition and their affective state, we conducted a Wilcoxon signed-rank test, comparing within-participant differences in their affective state.

To analyze Quiz 2 data, we computed frequencies and calculated accuracy percentages. An one-way ANOVA was performed to assess if children were more likely to correctly identify specific emotions when faces were partially covered with masks. Comparisons were made between the three aforementioned groups. Moreover, a Confusion matrix is used for evaluating children’s performance. A *post hoc* analysis using Scheffe criteria compared differences in groups. We also performed within-group repeated measures *t*-tests and error analysis to explore mood priming effects. Furthermore, a within-group, repeated measures *t*-tests were carried out to check for within-group differences. Last but not least, an error analysis reported to find the pattern of results and explanations of mood priming effects. We used an alpha level of 0.05 for all statistical tests.

## 3 Results

### 3.1 Facial emotion recognition

Descriptive Statistics were used to describe our data and then inferential statistics to generalize under two conditions (Table 1). The results of the participants’ face recognition performance indicate that children exhibit increased accuracy in facial emotion recognition in the non-masks condition. Our findings suggest that children can also accurately recognize “masked” anger, happiness, sadness, fear, and emotional neutrality from facial expressions that are partially covered by a medical, non-medical, surgical or cloth mask.

**Table 1:** Facial emotion recognition in masked and unmasked faces.

Inferential Statistics						
Facial Emotion	Anger	Fear	Sadness	Disgust	Neutrality	Happiness
*N*	69	69	69	69	69	69
Mask Condition Accuracy %	96%	92%	83%	59%	92%	97%
NO Mask Condition Accuracy %	99%	94%	90%	88%	94%	100%

Specifically, the one-sample binomial test resulted in significant *p*-values for happiness (97%, *p* = 0.000), sadness (83%, *p* < 0.001), anger (96%, *p* = 0.000), fear (92%, *p* = 0.000), and emotional neutrality (92%, *p* = 0.000), indicating that these emotions were recognized with high statistical confidence. However, for disgust (59%), the *p*-values were *p* = 0.050 and *p* < 0.001, respectively, suggesting that recognition of disgust was significantly lower but still above chance.

Moreover, an exact binomial test with exact Clopper–Pearson 95% CI was run on all students to determine if recognition accuracy of “masked” anger, fear, sadness, happiness, and emotional neutrality is considered more statistically significant than recognition accuracy of disgust (Table 2). Of the 69 children, only 41 (59%) correctly identified the emotion of disgust that had a 95% CI of 50 to 67%, *p* = 0.05.

**Table 2:** Binomial test and Clopper–Pearson 95% CI.

Confidence Interval Summary—Disgust				
Confidence Interval Type	Parameter	Estimate	95% Confidence Interval	
			Lower	Upper
One-Sample Binomial Success Rate (Clopper–Pearson)	Probability (disgust = right)	0.587	0.500	0.670
One-Sample Binomial Test Summary				
Total *N*	69			
Test Statistic	81.000			
Standard Error	5.874			
Standardized Test Statistic	1.958			
Asymptotic Sig. (2-sided test)	0.050			

Overall, the results from the exact Clopper–Pearson 95% CI indicate that children aged 6–7 years old, and not just the 69 sampled, are not accurate enough (59% accurate) to recognize disgust in faces that are partially covered by a medical, non-medical, surgical or cloth mask. Unfortunately, this means that we cannot be confident that children can accurately recognize the “masked” emotion of disgust.

To assess the relationship between children’s emotion recognition accuracy of basic emotions and their gender, we also used the Pearson Chi-Square statistic. However, our findings did not reveal any statistically significant differences between boys and girls. The *p*-values for all these analyses exceeded the predetermined alpha level of 0.05 (anger*gender: *p* = 0.659, happiness*gender: *p* = 0.175, fear*gender: *p* = 0.095, disgust*gender: *p* = 0.286, sadness*gender: *p* = 0.678, and emotional neutrality*gender: *p* = 0.81), indicating that gender did not appear to influence children’s ability to recognize these basic emotions.

### 3.2 Correlation between facial emotion recognition accuracy and children’s affective state

#### 3.2.1 Affective priming effect in groups

The correlation between facial emotion recognition accuracy and children’s affective state was also explored. Affective priming has been previously performed to investigate whether influencing students in a mood-congruent manner is effective with a specific focus on the targeted affective state for each group (Group A: Mood priming—Happiness, Group B: Mood priming—Sadness, Group C: Mood priming—Emotional Neutrality). A non-parametric Wilcoxon signed-rank test was conducted to compare within participants’ differences in affective state; the AffectLecture responses of each participant, before and after the affective priming, were used for this comparison. The affective priming main effect was significant. Children felt significantly happier after happy affective priming (*Z* = −3.946, *p* < 0.001), sadder after sad affective priming (*Z* = −4,176, *p* < 0.001) and neither happy nor sad after neutral affective priming (*Z* = −2,236, *p* = 0.025) (Table 3).

**Table 3:** Children’s pre-post affective state.

Test Statistics^a^		
Group (Before–After Affective Priming)	*Z*	Asymp. Sig. (2-tailed)
Group A: MoodPriming – Happiness	−3.946^b^	*p* < 0.001
Group B: MoodPriming—Sadness	−4.176^b^	*p* < 0.001
Group C: MoodPriming—Emotion Neutrality	−2.236^b^	*p* = 0.025

a Wilcoxon Signed Ranks Test.

b Based on negative ranks.

#### 3.2.2 Emotion recognition accuracy in groups

The study also examined whether there was a potential correlation between children’s affective state and their accuracy in recognizing the emotions of happiness, sadness, and emotional neutrality. Using the data from Quiz 2, Frequencies were computed, and performance was calculated as a percentage of correct responses. Upon closer analysis, Group A (Mood priming—Happiness) participants showed a strong 90.5% accuracy in recognizing happiness but struggled with sadness and neutrality, achieving a lower 61.9% overall accuracy, with 66.7% identifying sadness. While 88.5% of Group B (Mood priming—Sadness) participants accurately identified sadness, a notable challenge was evident as only 23.1% managed to correctly differentiate neutrality, yet 76.9% successfully recognized happiness. Group C (Mood priming—Emotional Neutrality) participants excelled in recognizing happiness (95.5%) and emotional neutrality (81.8%) but had a comparatively lower success rate in identifying sadness (68.2%) (Table 4).

**Table 4:** Emotion recognition accuracy across the three groups.

Statistics				
Group		Happy faces	Sad faces	Emotional neutrality
Group A: MoodPriming—Happiness	*N*	21	21	21
	Accuracy %	90.5%	66.7%	61.9%
Group B: MoodPriming—Sadness	*N*	26	26	26
	Accuracy %	76.9%	88.5%	23.1%
Group C: MoodPriming—Emotional Neutrality	*N*	22	22	22
	Accuracy %	95.5%	68.2%	81.8%

In summary, the study’s findings shed light on the varying degrees of accuracy in recognizing different emotions across the three groups. When it comes to identifying happy emotions, Group A: MoodPriming—Happiness exhibited the highest accuracy, with an impressive rate of 90.5%. Group C: MoodPriming—Emotional Neutrality also performed well, achieving an accuracy rate of 95.5% in recognizing happiness. However, for Group B: MoodPriming—Sadness, the accuracy in recognizing happy emotions was notably lower, standing at 76.9%.

On the other hand, when it came to recognizing sad emotions, Group B (MoodPriming—Sadness) excelled with an accuracy rate of 88.5%, while Group C (MoodPriming—Emotional Neutrality) achieved an accuracy rate of 68.2%. Group A (MoodPriming—Happiness) had the lowest accuracy in recognizing sadness at 66.7%. In identifying neutrality, Group C (MoodPriming—Emotional Neutrality) unsurprisingly exhibited the highest accuracy, with an accuracy rate of 81.8%. Notably, Group B (MoodPriming—Sadness) had the lowest accuracy in this category at 23.1%, while Group A (MoodPriming—Happiness) performed higher at 61.9%.

Following the results of the emotion recognition accuracy in masked faces, participants seem to make some wrong predictions on one of these affective states, e.g., on happiness. Thus, a 3 × 3 confusion matrix for the three classes was conducted to provide more information about children’s performance, offering insights into true positives, true negatives, false positives, and false negatives, aiding nuanced analysis beyond basic accuracy (Table 5). Quiz 2 comprised 12 questions, each associated with a corresponding picture and within these questions, there were four (4) images for each emotion, given 828 responses (happiness, sadness, and emotional neutrality).

**Table 5:** Confusion matrix.

Actual				
*n* = 828		Happy faces	Sad faces	Emotional neutrality
Predicted	Happy faces	TP = 240	FP = 20	FP = 40
	Sad faces	FN = 12	TP = 208	FP = 88
	Emotional neutrality	FN = 24	FN = 48	TP = 148

Additionally, one-way ANOVA of the percentage of accuracy on each emotion by group revealed that the groups did not differ significantly on happiness (*F*(1.997) = 1.55, *p* = 0.144) or sadness (*F*(1,960) = 0.28, *p* = 0.149), but they did differ significantly on emotional neutrality (*F*(11,100), *df*(2), *p* < 0.01). A *post hoc* Scheffe’s test on this result indeed confirmed that this was due to the emotional neutrality being significantly less identified than either of the other two emotions, who did not differ that much from each other. Specifically, Group A (MoodPriming—Happiness) indeed did not differ from Group B (MoodPriming—Sadness) on emotional neutrality recognition accuracy; both groups cannot be accurate about the recognition of emotionally neutral faces (*p* = 0.015).

#### 3.2.3 Within-group emotion recognition accuracy

Furthermore, a within-group, repeated measures *t*-test revealed a small but statistically significant difference in the recognition of happiness compared to sadness within Group A (MoodPriming—Happiness, *p* = 0.048). A *t*-test also indicated the significant correlation between recognition accuracy of happiness and emotional neutrality and children’s positive affective state (*p* = 0.010).

Similar *t*-tests were carried out to check for other within-group differences; within Group B (MoodPriming—Sadness) there are significant errors not only between the emotions of happiness and emotional neutrality (*p* < 0.001) but also between sadness and emotional neutrality (*p* < 0.001). No significant differences related to gender were observed.

An error analysis revealed that Group A (MoodPriming—Happiness) tends to confuse the emotion of sadness with the neutral emotion and the neutral emotion with the emotion of sadness likewise. On the other hand, Group B (MoodPriming—Sadness) more often confuses the neutral emotion with the emotion of sadness. The pattern of results and explanations of affective priming effects was also reviewed. As outlined above, a positive affective state had a significant effect on sadness recognition accuracy (*p* < 0.001). On the contrary, participants in a negative affective state after viewing the movie clip identify the “masked” neutral emotion as sadness (*p* < 0.001). The Group C (MoodPriming—Emotional Neutrality) did not think likewise, within-group tests did not report statistically significant performance decrements in emotion recognition.

Most significantly, results showed a negative bias for children in negative affective state and a positive bias for those in positive affective state. Particularly, following both positive and negative affective state priming promoted systematic inaccuracies in the perception of neutral affective state; a child that claims to be experiencing positive emotions not only enhanced its accuracy in recognizing that state, but also tends to label others’ emotions as positive too. Likewise, negative affective states tend to confuse children as they mistakenly label neutral emotion as negative, such as sadness.

## 4 Discussion

### 4.1 Emotion recognition in masked and unmasked faces

This study sheds light on the impact of face masks on children’s emotion recognition accuracy of basic emotions (anger, happiness, fear, disgust, sadness) and emotional neutrality, emphasizing the crucial significance of considering their affective state. Contrary to initial concerns and previous research (Spitzer, 2020; Bani et al., 2021; Carbon and Serrano, 2021; Gori et al., 2021; Levitan et al., 2022), findings have shown no evidence that face masks impair children’s ability to accurately discern emotions, even though recognizing disgust was more challenging. Within this framework, anger, happiness, fear, sadness, and emotional neutrality that are partially covered by a medical, non-medical, surgical or cloth mask are visible, recognizable, and, most importantly, accurately identifiable from children. However, children are not statistically accurate to recognize “masked” disgust. Stemming from these results, other authors predicted that happiness (Kastendieck et al., 2023) and disgust are less likely to be recognized in masked faces (Spitzer, 2020). According to the literature the most probable reason for this is that disgust is expressed primarily by the lower part of a face (Wegrzyn et al., 2017). Consistent with the literature (Widen and Russell, 2013; Gagnon et al., 2014), the most common errors made by school-age children consist in confusing disgust expressions; children are able to recognize disgust from a single facial action unit, nose wrinkle. Indeed, the case of disgust is importantly unique among basic emotions because it is the only one that can be expressed by a single facial action unit. All the other prototypical facial expressions theoretically associated with emotions include two or more facial action units (Gagnon et al., 2014).

The originality of our study from previous research may be attributed to several factors. Unlike Carbon’s study (2020), which focused on adults, our study specifically addresses children’s emotion recognition abilities, which may differ due to their developmental stage and adaptive nature. Additionally, our emphasis on considering children’s affective state, along with the unique configurations of micro-motor movements in their faces, contributes to the comprehensive understanding of how children perceive and interpret emotions even when masked. Furthermore, the analysis did not reveal any statistically significant differences in the recognition of basic emotions between boys and girls indicating that gender does not appear to be a significant factor in children’s accuracy in recognizing these emotions. Our findings also suggest that children may be quite successful in ignoring irrelevant visual information while focusing on reading affective states from the upper face and eyes only. As previously mentioned, participants displayed inconsistencies in recognizing others’ emotions, particularly in identifying “masked” anger, fear, and emotional neutrality. Guarnera et al. (2015) have observed a superior identification of emotional neutrality when the mouth area is presented, relative to the entire face or eye region. While we concur with the general consensus regarding the inherent complexity in identifying emotional neutrality, as supported by Guarnera et al. (2015) and Carbon (2020), we believe that there is presently an inadequacy of empirical evidence to support that “masked” emotional neutrality can be reliably identified in the context of child participants. This was a hypothesis we indeed anticipated would pose a significant challenge.

We also found that “masked” anger is absolutely identifiable from children. The robustness of anger perception is supported by findings (Wegrzyn et al., 2017) showing that the upper part of a face is essential for the recognition of anger and fear. Reading “masked” sadness was particularly challenging for children. There seems to be general agreement. A study by Guarnera et al. (2015) demonstrated that sadness was best identified when the eye area was presented, followed by presentation of the whole face. Wegrzyn et al. (2017) also discussed that the lower part of a face is essential for the recognition of happiness, the upper portion for anger and fear, and both for surprise and sadness. Where confusions arise, between fear and surprise, and between disgust and anger, for instance, there are likely to be implications for social interaction; for example, a child may behave inappropriately, leading to misunderstanding and conflict.

### 4.2 Correlation between facial emotion recognition accuracy and children’s affective state

While there is a great deal of research on facial expression recognition, the study of normal variation of affective state on emotion recognition in children is scarce. No studies have investigated how children’s affective state is related to emotion recognition. Our study explored the correlation between emotion recognition accuracy and children’s current affective state by implementing affective priming. The results are discussed in terms of the significant role of children’s current affective state in their recognition of emotion in others; labeling others as experiencing, or not experiencing, particular emotions.

For children in positive affective states, a positive bias emerged; children in positive affective states obviously recognize happiness more accurately than emotional neutrality and sadness. The results align with the claims of Gasper (2004) who identified that one’s own emotional experiences are an important source of information to understand how another person is feeling. Based on the findings of similar studies, positive moods facilitate recall of positive stimuli and making positive judgments about others (Drace et al., 2010). Recently, Trilla et al. (2021) investigated in the same way that adults project their own affective states when reading other’s emotional expressions; facial expressions were more readily classified as happy when participants reported feeling happy. They also found that participants were more likely to classify facial expressions as sad when they reported feeling sad themselves, likewise (Trilla et al., 2021). The data suggest that for children in negative affective states, a negative bias emerged—sad participants recognize sadness more accurately than happiness and emotional neutrality. This is also consistent with findings from Voelkle et al. (2014), which demonstrated a clear mood-congruency effect in emotion perception. They found that participants in a more positive mood were more likely to perceive happiness in presented faces, whereas higher levels of negative mood were associated with a decreased probability of perceiving additional happy expressions. Furthermore, Terwogt et al. (1991) found intriguing results regarding the influence of mood on the labeling of expressions. In their study, children in a happy mood condition tended to overuse the label “happy” compared to those in control or sad conditions. However, the effects of mood on labeling were not symmetrical, as there was only a trend for sad children to label ambiguous expressions as “sad,” which did not reach significance.

Most importantly, children both in positive and negative affective states did not differ significantly on recognizing happiness or sadness, but they did differ significantly on emotional neutrality. To the best of our knowledge, no previous research has investigated whether children promoted systematic inaccuracies in the perception of neutral affective state. However, based on the findings of similar studies, a more plausible explanation is that a child that claims to be experiencing positive emotions not only enhances its accuracy in recognizing that state, but also tends to label others’ emotions as positive too. Likewise, children in negative affective states tend to mistakenly label neutral emotion as negative.

From another perspective, when it comes to identifying happiness, children in positive affective states exhibited the highest accuracy; that contributes to a clearer understanding of what positive bias means. On the other hand, when it came to recognizing sad emotions, children in negative affective states achieved the best accuracy rate. As a rule, happy children will more accurately recognize happy affective states than sad, whereas sad children will better recognize negative affective states. It is also worth mentioning that, in line with Chepenik et al. (2007), our results revealed a general decline in emotional neutrality recognition performance. In identifying neutrality, only children in neutral mood unsurprisingly exhibit high accuracy. Therefore, an important issue is that children in negative affective states had the lowest accuracy in the recognition of emotionally neutral faces. In conclusion, this theme persists in the focus of attention not only beyond the pandemic but also in the foreseeable future, whenever and wherever masks should be mandatory for children, as it is always important to be aware of one’s capacity to adapt to non-verbal communication.

In children 6 and 7 years old Stegge et al. (1995) found a symmetrical mood induction; happy children labeled more drawings as happy, compared to sad children. This symmetrical mood congruity effect is consistent with the theoretical notions of the “mood repair” hypothesis, which states that the asymmetrical mood effects (i.e., more pronounced effects of a happy that of a sad mood) that are often found in adults will be absent in young children, as they have not yet learned the strategies necessary to counteract the consequences of a negative feeling state (Isen, 1984).

In essence, our study suggests that children’s selective attention and judgments are influenced by their affective state, although other factors may also be at play. While we aimed to induce affective states through exposure to scenes from the Pixar movie, it’s possible that the observed results were influenced by the suggestive nature of the manipulation rather than genuine mood induction. Specifically, the observation that the group in neutral priming identified happy and neutral emotions more frequently than sad faces raises questions about the role of context in shaping participants’ responses. While we maintain our hypothesis that affective states influence facial expression recognition accuracy, we acknowledge the need to explore the potential impact of contextual factors on participants’ interpretations. Unfortunately, our study does not allow us to clearly disentangle the underlying mechanisms in terms of mood priming vs. emotion elicitation. While we stand by our hypothesis that affective states influence facial expression recognition accuracy, we acknowledge the need to explore the potential impact of contextual factors on participants’ interpretations. This does not automatically imply that the effects we are studying are weak. What it does show, however, is that mood congruity in children is a rather complex phenomenon connected to other psychological processes or the broader contextual cues present during the experimental task. Nonetheless, some intriguing questions remain unanswered by this study and merit exploration in future research. For instance, does a child’s attention lean more towards positive stimuli, even when experiencing a sad mood? Furthermore, an intriguing inquiry lies in whether children derive greater happiness from interacting with individuals whose facial expressions align with their own affective state.

## 5 Limitations

Several factors need to be considered upon understanding the limitations of this study. It is crucial to recognize that the scope of this investigation was focused on a specific set of basic emotions and a specific age group (children). Consequently, it is essential to exercise caution when applying findings to a broader population or a wider range of emotions. At the same time, further research could explore more diverse samples and additional emotions to provide a more comprehensive understanding of the role of gender in emotion recognition. In addition, the adult stimuli were obtained from a pre-existing database, but it is essential to consider that these emotions may not represent spontaneous, genuine expressions. Depicted emotions may not fully generalize to spontaneous, authentic “real” expressions. One significant limitation worth noting is the absence of contextual information in this study. Emotions are typically not observed in isolation; they are often intertwined with the environment and social context, providing crucial additional cues for recognition. Existing literature emphasizes the substantial influence of social contexts on emotion recognition. Consequently, the absence of contextual information in our study highlights the need for future research to explore the influence of environmental and social contexts on emotion recognition more extensively.

## Data availability statement

The raw data supporting the conclusions of this article will be made available by the authors, without undue reservation.

## Author contributions

MM: Data curation, Investigation, Methodology, Formal Analysis, Writing – original draft, Writing – review & editing. SK: Data curation, Investigation, Methodology, Writing – original draft, Writing – review & editing. ID: Data curation, Methodology, Writing – original draft, Writing – review & editing. PDB: Conceptualization, Funding acquisition, Project administration, Supervision, Writing – original draft, Writing – review & editing.
