# Supplementary material for: Masked emotions: does children’s affective state influence emotion recognition?
Source: Front Psychol. 2024 Jun 19;15:1329070. doi: 10.3389/fpsyg.2024.1329070 (PMC11220387; doi:10.3389/fpsyg.2024.1329070)
Supplement: Supplementary file 1 [file Table_1.docx]

Supplementary Material

**Supplement 1**

| **Tests of Normality**  **Shapiro – Wilk Test** | | | | | | | |
| --- | --- | --- | --- | --- | --- | --- | --- |
| **Masked Emotions** | | | | **Unmasked Emotions** | | | |
|  | Statistic | *df* | Sig. |  | Statistic | *df* | Sig. |
| anger | .099 | 69 | <.001 | anger | .207 | 69 | <.001 |
| fear | .248 | 69 | <.001 | fear | .346 | 69 | <.001 |
| sadness | .346 | 69 | <.001 | sadness | .477 | 69 | <.001 |
| disgust | .372 | 69 | <.001 | disgust | .608 | 69 | <.001 |
| neutrality | .248 | 69 | <.001 | neutrality | .317 | 69 | <.001 |
| happiness | .436 | 69 | <.001 | happiness | .539 | 69 | <.001 |
| a. Lilliefors Significance Correction | | | | | | | |
